# Efficient degassing and ppm-level oxygen monitoring flow chemistry system†

**DOI:** 10.1039/d3re00109a

**Published:** 2023-04-28

**Authors:** Paulius Baronas, Jacob Lynge Elholm, Kasper Moth-Poulsen

**Affiliations:** a The Institute of Materials Science of Barcelona, ICMAB-CSIC Bellaterra 08193 Barcelona Spain; b Catalan Institution for Research & Advanced Studies, ICREA Pg. Lluís Companys 23 08010 Barcelona Spain; c Chalmers University of Technology, Department of Chemistry and Chemical Engineering SE-412 96 Gothenburg Sweden; d Department of Chemical Engineering, Universitat Politècnica de Catalunya, EEBE Eduard Maristany 10-14 08019 Barcelona Spain kasper.moth-poulsen@upc.edu https://www.moth-poulsen.com

## Abstract

Low oxygen levels are critical for a long range of chemical transformations carried out in both flow and batch chemistry. Here, we present an inline continuous flow degassing system based on a gas-permeable membrane inside a vacuum chamber for achieving and monitoring ppm-level oxygen concentrations in solutions. The oxygen presence was monitored with a molecular oxygen probe and a continuously running UV-vis spectrometer. An automated setup for discovering optimal reaction conditions for minimal oxygen presence was devised. The parameters tested were: flow rate, vacuum pressure, solvent back-pressure, tube material, tube length and solvent oxygen solubility. The inline degassing system was proven to be effective in removing up to 99.9% of ambient oxygen from solvents at a flow rate of 300 μl min^−1^ and 4 mbar vacuum pressure inside the degassing chamber. Reaching lower oxygen concentrations was limited by gas permeation in the tubing following the degassing unit, which could be addressed by purging large volume flow reactors with an inert gas after degassing or by using tubing with lower gas permeability, such as stainless steel tubing. Among all factors, oxygen solubility in solvents was found to play a significant role in achieving efficient degassing of solvents. The data presented here can be used to choose optimal experimental parameters for oxygen-sensitive reactions in flow chemistry reaction setups. The data were also fitted to an analytically derived model from simple differential equations in physical context of the experiment.

## Abstract 2

A method for in-line continuous flow degassing to remove up to 99.9% of oxygen from solvents is presented.

## Graphical abstract

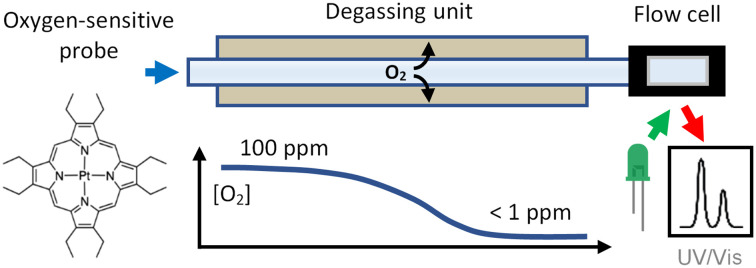

## 1 Introduction

Recent advances in continuous-flow systems show their large potential in the automation of chemistry and chemical analyses.^1–8^ By utilising continuous-flow platforms, computer-assisted screening of the photophysical properties of molecular probes can significantly accelerate reaction discovery.^9–11^ This shows the importance of developing continuous-flow analogues of experimental techniques that are currently used in regular batch processing.

High oxygen reactivity and its concentrations in ambient air require measures to degas solvents and solutions for some chemical reactions or to degas samples for spectroscopic characterisation. Reducing oxygen contents in sensitive reactions, such as metal-mediated cross-coupling, could offer substantial material savings as well as change the outcome of reactions.^12,13^ Similarly, the characterisation of organic molecules whose emission efficiency relies on triplet energy levels requires degassing to remove oxygen that acts as a triplet energy quencher.^14^ While there are multiple methods to achieve ppm-level oxygen concentrations in batch processing *e.g.* inertisation manifolds, inert gas purging or freeze–pump–thaw techniques, inline oxygen removal in continuous-flow systems can be more challenging.

Oxygen removal in flow can be based on oxygen-scavenging or membrane-based degassing systems. Micro-scale oxygen degassing chips produced from oxygen-scavenging polymers have been shown to reduce oxygen concentrations down to 0.4% (removing 99.8% of ambient oxygen) in passing solutions.^14,15^ Unfortunately, oxygen-scavenging microchips are not ideal for the microliter to milliliter volume scales that most flow chemistry and analysis systems are operating at. In such flow systems, degassing as well as gas–liquid mixing can be achieved by using membranes with high gas permeabilities. For example, Teflon AF-2400 has an oxygen permeability of 960 barrers, which is hundreds of times higher than that of other typical fluoropolymers.^16^ Therefore, the Teflon AF-2400 polymer is a popular choice of material to introduce gases in tube-in-tube reactors.^17–29^ Similarly, Teflon AF-2400 membrane reactors can also be used for the degassing of a solution, where the oxygen concentration is regulated by the vacuum level in the degassing chamber and the flow rate of the liquid.^30^

**Fig. 1:**
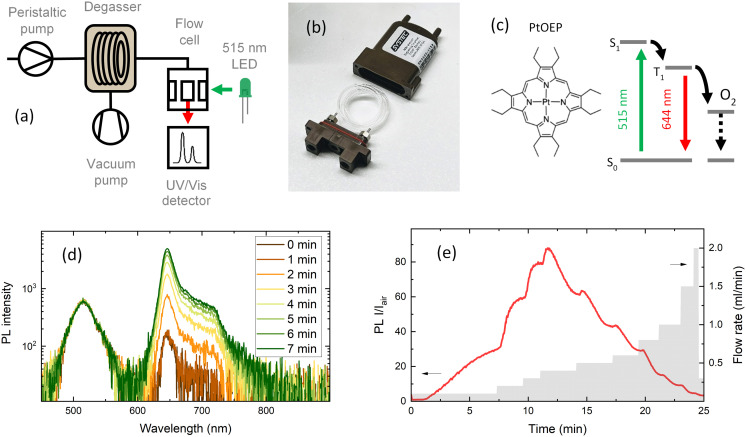
Method for oxygen sensing in continuous flow. (a) Schematic of liquid degassing in continuous flow. Measurements of PL intensity are performed in a flow cell. (b) A commercial degassing unit with oxygen-permeable membrane tubing in a vacuum chamber. (c) Phosphorescent PtOEP compound dissolved in a 5 μM toluene solution with emission at 644 nm when excited with 515 nm light. Emission is suppressed under saturated oxygen conditions due to triplet energy transfer from the PtOEP triplet state to O_2_, resulting in non-radiative losses. (d) Increase in PL intensity at a constant flow rate of 0.1 ml min^−1^ and 4 mbar vacuum chamber pressure. (e) Integrated photoluminescence intensity ratio (*I*/*I*_air_) under degassed and air saturated conditions recorded at different PtOEP solution flow rates. The flow rate was increased after steady-state was reached.

In this paper, we present a flow chemistry-based automated setup for discovering optimal conditions (*i.e.* flow rate, vacuum pressure, solvent gas solubility) for minimal oxygen concentration. The oxygen concentration was monitored using the photoluminescence (PL) quenching of an oxygen-sensitive molecular probe. Fig. 1a shows the scheme used to degas and monitor the oxygen concentration in solution. Inline oxygen removal was performed by using a commercial mini degassing unit with gas permeable tubing (Fig. 1b), which is generally used to remove air bubbles for improving HPLC instrument accuracy. The system is designed to operate at a pressure of 60 mbar; however, as we show here, the degassing unit showed reliable performance for oxygen removal at lower than recommended vacuum pressures. Under optimised conditions, this technique allowed us to achieve a level of oxygen below the ppm level (removing up to 99.9% of the molecular oxygen) present in the solution exposed to atmospheric air. The degassing setup was thoroughly tested to find the optimal parameters for reducing the oxygen level. The parameters tested were: flow rate, vacuum pressure, tube material and tube length. Different tube materials have different gas permeabilities,^16^ which impact the reabsorption of atmospheric gases in the tubes between the degassing unit and the PL flow cell. Investigating the flow rate impact showed two opposite effects. With slower flow rates, the residence time of the solution within the tube increases, and therefore the reintroduction of atmospheric gases increases. To limit the reintroduction of gases, the residence time within the tube should be as short as possible to keep the oxygen concentration as low as possible. However, the degassing efficiency within the mini vacuum unit increases with longer residence times, that is, with slower flow rates. The task is then to find the optimal flow rate that satisfies a long enough residence time for degassing and a short enough residence time to limit the reabsorption of gases.

## 2 Experimental setup

A molecular oxygen probe platinum(ii) octaethylporphyrin (PtOEP) was purchased from Sigma-Aldrich and used as received. Tubes of perfluoroalkoxy alkanes (PFAs) and Tefzel™ ethylene tetrafluoroethylene (ETFE) were purchased from IDEX. Tubing pieces of 0.04′′ (1 mm) internal diameter (I.D.) and 1/16′′ (1.56 mm) external diameter (O.D.) were used in all experiments. A mini degassing unit with a 925 μl internal tube volume (Fig. 1b) was purchased from IDEX. The dimensions of the Teflon AF-2400 tubing inside the degassing unit were 0.04′′ I.D. and 1/16′′ O.D. A simplified scheme of the flow setup and fluorescence detection system is shown in Fig. 1a. The liquid flow in the system was provided by peristaltic pumps (Vapourtec V-3) integrated into a flow chemistry module (Vapourtec R-Series). An absolute vacuum pressure inside the degassing chamber in the range of 4 to 60 mbar of atmospheric air was achieved with a membrane vacuum pump (Buchi V-300) and monitored with an evaporation system (Buchi Rotavapor R-300). A minimum of 0.5 mbar pressure inside the degassing chamber was reached using a dual-stage rotary vane mechanical vacuum pump (Edwards RV3). The vacuum pressure was measured with an electronic vacuum gauge (Vacuubrand Vacuu-View extended). The fluorescence signal was measured in flow through a fluorescence cell with an internal volume of 100 μl (Starna Cells Type 583F). The flow cell was placed in a sample holder with fiber coupling (Avantes CUV-ALL/UV/VIS). A 515 nm LED light source (Thorlabs M530L4) coupled to a 400 μm optical fiber (Avantes FC-UVIR400) was used for excitation. The fluorescence was detected with a UV-vis spectrometer (Avantes AvaSpec-ULS2048CL-EVO-RS-UA). The high detection sensitivity needed for weak emission was achieved by placing a 200 μm slit and using a 400 μm optical fiber. A 10 ml stainless steel reactor with 1 mm I.D. tubing was purchased from Vapourtec. A commercial oxygen gas sensor (MBRAUN MB-OX-SE1) connected to a glovebox system was used to calibrate the oxygen concentration in the degassed solution. A Python program was written to control the flow rates of a set of Vapourtec pumps and the detection of the fluorescence signal from the Avantes spectrometer using the OPC-UA standard and Python API examples provided by the manufacturer. The code repository can be found here (https://github.com/Elholm/KMP-Group).

## 3 Results and discussion

### 3.1 Photoluminescence quenching-based oxygen concentration measurements

Among the methods to determine oxygen levels, optical sensing is advantageous due to the possibility of remote sensing.^31^ The majority of commercial optical oxygen sensors are based on the measurement of PL quenching of a molecular probe by oxygen. Here, the probe material is in a solution, which is excited with LED light and emits a red-shifted PL with an intensity inversely proportional to the oxygen concentration. One of the most explored classes of materials for oxygen probes is metal–ligand complexes. The long excited state lifetime of metal–ligand complexes extends the limits of oxygen detection, resulting in PL ratios extending over several orders of magnitude when compared to those under hypoxic and oxygen-saturated conditions. In this work, PtOEP was selected as the oxygen sensing probe, which shows a broad absorption band in the 470–550 nm region and a significantly red-shifted emission centered at 644 nm (Fig. 1c and d). In PtOEP, the excitation of the singlet state S_1_ is followed by ultrafast intersystem crossing, where phosphorescence occurs from the lowest triplet state T_1_.^32^ In an oxygen-saturated environment, phosphorescence quenching is induced by energy transfer from the triplet state to molecular oxygen. This results in a low phosphorescence quantum yield when the PtOEP solution is exposed to ambient air and a significant increase in emission intensity upon degassing of the solution. Bansal *et al.* reported the phosphorescence quantum yield in an air-saturated PtOEP toluene solution to be 0.125% which increased up to 40.15% after 2 hours of ultrapure nitrogen gas purging, resulting in an increase in PL intensity of 320 times.^32^

The oxygen content in a degassed PtOEP solution was quantified by the Stern–Volmer equation that describes the quenching process in a homogeneous system:^33,34^1
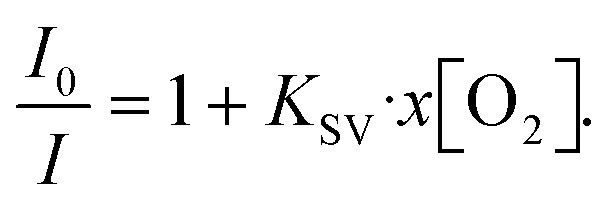


Here, *I*_0_/*I* is the ratio of PL intensities in the absence of O_2_ (*I*) and at oxygen concentration *x*[O_2_] (*I*_0_), and *K*_SV_ is the Stern–Volmer constant. The ratio *I*_0_/*I*_air_ was measured by degassing the PtOEP solution in a glovebox with a known O_2_ concentration of 2.4 ppm and comparing its PL intensity to that of an air-saturated sample where an ambient oxygen level of 20.95% was assumed. The true concentrations of oxygen in solution at room temperature were determined using Henry's law:2
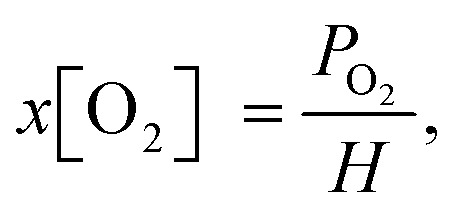
where *x* is the mole fraction of oxygen and *P*_O_2__ is the oxygen pressure. A Henry's constant *H* of 1050 bar for oxygen solubility in toluene at room temperature was obtained from the literature.^35^ The measured *I*_0_/*I*_air_ ratio was 309 and the Stern–Volmer quenching constant *K*_SV_ = 1.5 × 10^6^ was obtained for the oxygen quenching of PtOEP in toluene (for more details, see ESI† Fig. S1). By using eqn (1), the molar oxygen concentration in solution after degassing can be determined from the measured *I*/*I*_air_ ratio. Here, the high dynamic range of the PtOEP oxygen probe combined with highly accurate calibration using a commercial oxygen sensor allows for small errors (±0.001–3 ppm) for determining the oxygen concentration in solution (see Fig. S6 in the ESI†). The accuracy of the oxygen sensor increases at low oxygen concentrations, where *I*/*I*_air_ is the highest.

To evaluate the oxygen removal efficiency of the inline flow degassing system, a 5 μM solution of PtOEP in toluene was pumped through the degassing unit towards the flow cell (Fig. 1a). The degassing unit and flow cell were connected with a short (10 cm) PFA tube with an internal diameter of 1 mm and volume of 78.5 μl. The solution was excited with a 515 nm LED, and the resulting emission was recorded using a UV-vis spectrometer, which collected spectra every two seconds. Fig. 1d shows the increase in emission intensity of the 644 nm phosphorescence band of PtOEP after setting a 4 mbar vacuum pressure inside the degassing chamber at a steady solution flow rate of 100 μl min^−1^. Taking into account the large 925 μl tube volume inside the degassing chamber, the maximum degassing efficiency at 100 μl min^−1^ is reached after approximately 10 minutes.

Fig. 1e shows the emission intensity ratio recorded at the 644 nm emission maximum as a function of time (left *y*-axis), for every flow rate (right *y*-axis). The PL intensity ratio (*I*/*I*_air_) visualises how many times the emission intensity is increased in a degassed solution compared to that under air-saturated conditions. Interestingly, *I*/*I*_air_ as a function of flow rate shows that the highest degassing efficiency was recorded at intermediate flow rates of 300–400 μl min^−1^, suggesting that at long residence times, the degassing rate becomes slower than the reabsorption of gases in the tubing between the degassing unit and the flow cell. This shows that the gas permeability of the tubing following the degassing unit is an important parameter when aiming for low oxygen concentrations.

### 3.2 Effects of vacuum pressure inside the degassing chamber

**Fig. 2:**
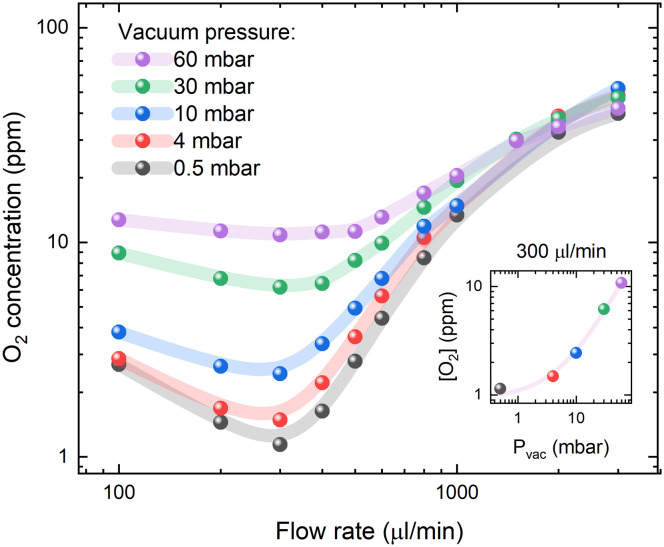
Oxygen concentration in PtOEP toluene solution at different flow rates and degassing unit vacuum pressure. The inset shows the minimum O_2_ concentration *versus* vacuum pressure for a 300 μl min^−1^ flow rate and a linear fit of the data points. PFA tubing of 10 cm length, 0.04′′ internal diameter and 1/16′′ external diameter was used between the degassing unit and the measurement cell.

According to Henry's law (eqn (2)), the oxygen concentration in solution under steady-state conditions is mainly influenced by the partial oxygen pressure in the environment. However, the dynamic movement of the liquid within the membrane tubing must lead to inefficient degassing at higher flow rates. The estimated O_2_ concentrations as a function of vacuum pressure inside the degassing chamber and solution flow rates are presented in Fig. 2. The highest degassing efficiency is reached at a flow rate of 300 μl min^−1^, while the oxygen concentration increases at slow flow rates due to oxygen reabsorption and at high flow rates due to insufficient degassing. Interestingly, at flow rates higher than 1 ml min^−1^, the vacuum pressure in the degassing chamber has minimal effects on the degassing efficiency, while the oxygen contents are still up to 10 times lower than the ambient oxygen concentration of [O_2_] = 200 ppm in toluene. This suggests that [O_2_] = 10–20 ppm oxygen concentrations in a 925 μl degassing unit can be achieved at a relatively short residence time (less than 1 minute) and low vacuum pressure, but lower O_2_ concentrations require a higher vacuum as well as longer residence times.

At a flow rate of 300 μl min^−1^, the decrease in vacuum pressure inside the degassing chamber from 60 to 4 mbar results in a similar magnitude decrease in oxygen concentration in solution. This is indicated by the linear relation shown in the inset of Fig. 2. Deviation from linear behaviour at vacuum pressure below 4 mbar indicates that oxygen reabsorption becomes significant; therefore, no lower oxygen concentration can be achieved by further reducing the vacuum pressure inside the degassing chamber. According to Henry's law (eqn (2)), oxygen concentration in toluene at 4 mbar vacuum pressure is equal to [O_2_] = 20.95%·(4 × 10^−3^ bar)/(1050 bar) = 0.8 ppm, which can be achieved at long residence times. The measured oxygen concentration in solution already approaches this value at a flow rate of 300 μl min^−1^. It is only limited by gas reabsorption in the tubing between the degassing unit and the fluorescence flow cell.

Another parameter that can be controlled in the flow system and could influence the degassing and gas reabsorption rates is the solvent back-pressure. To test this, a back-pressure regulator was connected after the fluorescence flow cell to create 5 bar of solvent pressure in the flow system. Oxygen concentration measurements were performed for short (10 cm) and long (100 cm) tubing between the degassing unit and the fluorescence cell to investigate both degassing efficiency and gas reabsorption in the tubing following the degassing unit (see Fig. S3 in the ESI†). However, when comparing oxygen concentrations at the different flow rates, only a marginal decrease in oxygen concentration was observed at slow flow rates (below 300 μl min^−1^) after the solution passed the short tube (10 cm) at 5 bar compared to that with no back-pressure. This could indicate that gas permeation into the solvent through tubing after the degassing unit occurs at a slower rate when back-pressure is applied.

### 3.3 Effects of tubing gas permeability on gas reabsorption

**Fig. 3:**
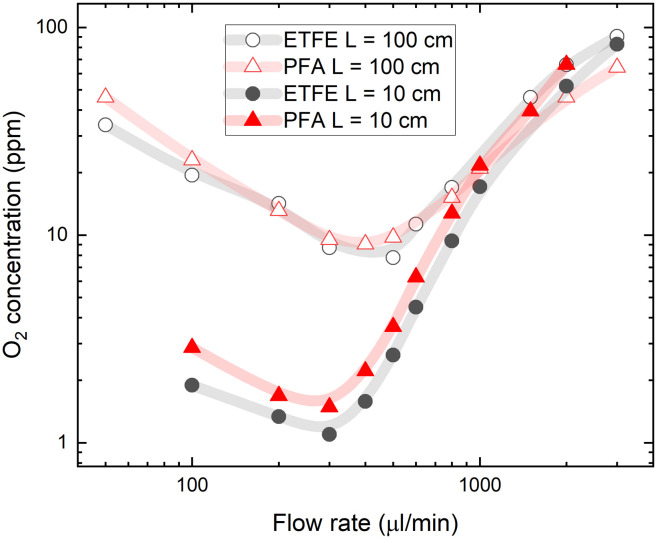
Oxygen concentration in PtOEP toluene solution as a function of tubing gas permeability and length. Oxygen concentration was measured after a single pass of PFA and ETFE tubing of 10 and 100 cm length at different solution flow rates. The vacuum pressure inside the degassing chamber was kept at a constant 4 mbar.

A practical challenge for carrying out flow chemistry reactions at low O_2_ concentrations can be oxygen reabsorption through the tubing walls, which happens downstream from the degassing unit. The gas permeability of tubes outside the degassing unit should be considered when aiming for a lower oxygen concentration in the flow system. To explore the effects of reabsorption of O_2_, we tested different tubing materials and lengths. For experiments shown in Fig. 2, PFA tubing material was used between the degassing unit and the flow cell with an oxygen permeability of 10.3 barrers.^36^ This resulted in significant gas reabsorption even in the short 10 cm PFA tube after the degassing unit. Lower gas reabsorption after the degassing unit can be achieved using ETFE fluoropolymer tubing, which has up to 10 times lower gas permeability.^37^Fig. 3 shows the O_2_ concentration measured in toluene after passing short (10 cm) and long (100 cm) PFA and ETFE tubes between the degassing unit and the flow cell. Despite the large difference in the gas permeability of the tubing materials, the measured oxygen concentrations are only slightly lower after passing the short ETFE tube compared to that of the same-length PFA tube.

In the longer, 100 cm tubing, the oxygen concentration in solution was increased almost 10-fold at a flow rate of 300 μl min^−1^ due to oxygen reabsorption given the longer residence time. Interestingly, for long tubes, the different gas permeabilities of PFA and ETFE did not influence the final oxygen contents measured at the flow cell. This shows that the majority of gas reabsorption occurs at the beginning of the tubing after the degassing unit, where the oxygen concentration difference (inside the tube relative to the ambient concentration outside the tube) is high enough to drive the permeation of oxygen through the tube walls. Oxygen reabsorption becomes irrelevant at high flow rates (above 1 ml min^−1^), which is reflected in converging oxygen concentrations for both tube materials and tube lengths (see Fig. 3).

These findings show that low oxygen concentrations are difficult to achieve in large volume reactors due to significant oxygen permeability *via* tubing downstream from the degassing unit. However, from the data presented here, predictions on oxygen levels in large-volume reactors can be made and will be presented in the section on computational modelling. Furthermore, other measures can be taken to reduce oxygen concentration by purging reactors with inert gases after degassing. This can be achieved with an inline tube-in-tube reactor purged with inert gases at low pressures to avoid bubble formation.^24^ A significant reduction in oxygen concentration was achieved by fitting 1 m of 1 mm I.D. PFA tubing inside 3 mm I.D. PVC tubing and purging the internal volume with a low pressure (40 mbar) of N_2_ gas (see Fig. S4 in the ESI†). This shows that inline degassing coupled with inert gas purging is a good strategy to reduce oxygen concentration.

### 3.4 Computational approach

In the literature, several methods have been studied to model the evolution of gas or solute concentration throughout an experiment based on differential equations.^38–41^ This particular system can be simulated using a relatively simple model to predict the oxygen concentration of the solvent at the measurement point. Based on the flow rate of the full system, the residence times within the vacuum degassing unit and the tubing can vary from seconds to minutes. The simulation starts at the beginning of the vacuum degassing unit and predicts the reduction in oxygen concentration based on the residence time calculated from the flow rate. The oxygen concentration gradient within the vacuum degassing unit is given as3
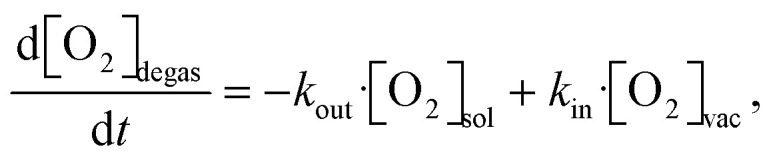
where *k*_out_ and *k*_in_ describe the rate constants for diffusion of oxygen through the tubing within the vacuum degassing unit, which is related to the oxygen permeability of the tubing material. The oxygen concentration gradient within the tubing after passing the degassing unit is given as4
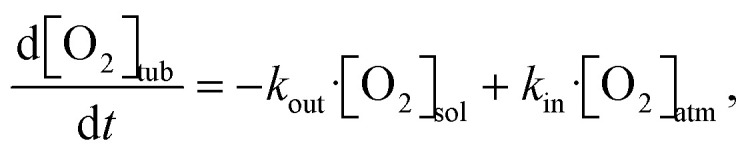
where *k*_out_ and *k*_in_ describe the rate constants for diffusion of oxygen through the tubing after the degassing unit. Now these simple differential equations have analytical solutions that are readily solved:

5 Degassing unit concentration: [O_2_]_degas_ = −*C*·exp(−*k*_out_·*t*) + *C*·[O_2_]_vac_

6 Tubing concentration: [O_2_]_tub_ = *C* − exp(−*k*_out_·*t*)

where *C* is the integration constant, determined to be the O_2_ concentration under ambient conditions readily computed using Henry's law. The final oxygen concentration in the solution is predicted by subtracting the reduction in concentration while in the vacuum degassing unit from the starting concentration of 21% (atmospheric concentration) and afterwards adding the increase in concentration while in the tubing before the measurement point.

**Fig. 4:**
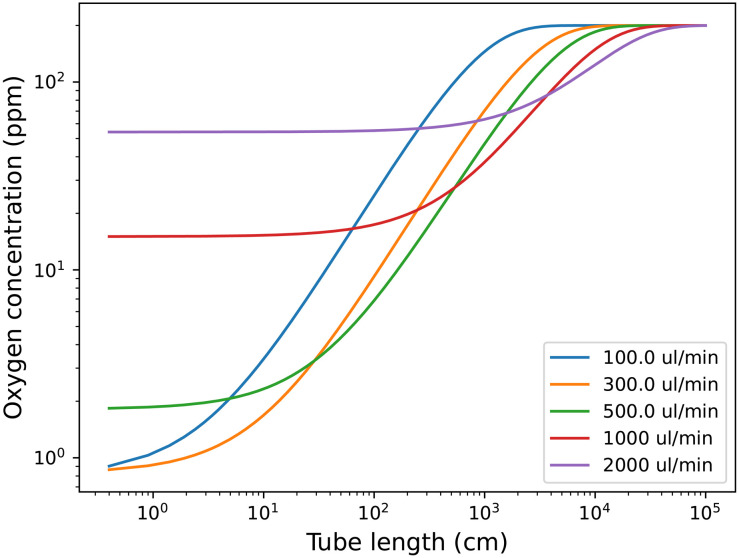
Prediction of oxygen level in toluene solution depending on tube length after the degassing unit with ETFE tubing (0.04′′ internal diameter and 1/16′′ external diameter) and a vacuum pressure of 4 mbar inside the 0.925 ml degassing unit. The starting concentration is the predicted oxygen level after the degassing unit at the given flow rate.

The rate constants were fitted to the experimental data, but they should be quite explainable with the chemical properties of the tubing material, *e.g.*, gas permeability. The fit for the analytical solution to the experimental data is available in Fig. S5 in the ESI.† The rate constants obtained from the fittings can be used to predict oxygen levels at other tube lengths and flow rates, as shown in Fig. 4. This can be used to predict which flow rate to use, at a relevant tube length (reactor volume) to obtain the lowest possible oxygen level in the solution.

### 3.5 Effects of solvent oxygen solubility

Another factor that could influence degassing properties is the oxygen solubility of the solvent used in the flow system. To test how oxygen solubility affects the degassing efficiency, oxygen concentrations in acetonitrile (MeCN) were also measured. Henry's constant for oxygen solubility in MeCN at room temperature is 2040 bar.^42^ This indicates a two times lower oxygen solubility compared to toluene (Tol). For the MeCN solution, the Stern–Volmer constants were recalculated by performing calibration using an oxygen-free solution degassed in the glovebox (for more details, see Fig. S2 in the ESI†).

**Fig. 5:**
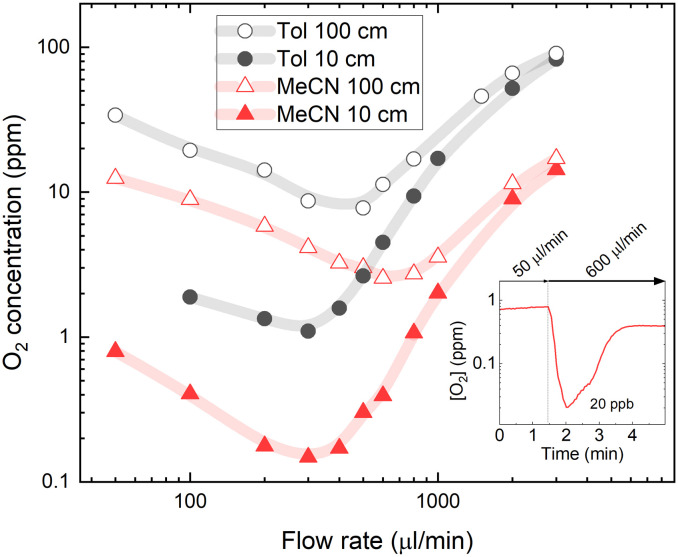
The oxygen concentration in PtOEP solution as a function of solvent oxygen solubility. The oxygen concentration was measured in toluene (Tol) and acetonitrile (MeCN) solvents after passing through 10 and 100 cm ETFE tubing at different flow rates. The vacuum pressure inside the degassing chamber was kept at a constant 4 mbar. The inset illustrates the temporary decrease of oxygen concentration in MeCN solvent after passing 10 cm ETFE tubing using a slow-to-fast flow rate sequence.

Fig. 5 shows estimated molar oxygen concentrations in 5 μM PtOEP solutions in MeCN and Tol. Note that the molar oxygen concentrations in MeCN appear twice as low as those in Tol due to the approximately two times smaller molecular weight of the former. Nevertheless, almost an order of magnitude lower O_2_ concentration in the 0.3–3 ml min^−1^ flow rate range measured after passing short (10 cm) ETFE tubing suggests highly efficient degassing associated with low oxygen solubility in MeCN. The higher degassing efficiency in MeCN is also supported by a slight shift of the lowest achieved oxygen concentration toward a higher flow rate, which shows that a shorter residence time inside the degassing unit is required to remove oxygen from the solvent. Higher degassing efficiency is particularly evident in reduced oxygen concentrations measured after passing the long (100 cm) ETFE tube that could also indicate slower oxygen reabsorption in MeCN solvent.

Interestingly, the recorded below ppm-level oxygen concentrations in MeCN were lower than the concentrations predicted by Henry's law at vacuum pressure in the degassing unit. After passing the short tubing at an optimal degassing flow rate of 300 μl min^−1^, the oxygen concentration in the MeCN solution was reduced to 0.15 ppm, which is lower than the 0.4 ppm predicted by Henry's law for a vacuum pressure of 4 mbar inside the degassing chamber. This suggests that oxygen diffusion from solution *via* membrane to the degassing chamber driven by an oxygen concentration difference may not be the only mechanism causing a degassing of the solvent. The possibility of solvent evaporation through the membrane inside the degassing chamber that would effectively increase the solution concentration was investigated by repeating experiments with a rhodamine B luminescent probe diluted in solvent with luminescence insensitive to oxygen content; however, no changes in solution concentration could be detected. This can be explained by the significantly lower permeability of solvent vapour through the tube membrane. Furthermore, even lower oxygen concentrations (down to 20 ppb) can be achieved by using a sequence of slow-to-fast flow rates (see inset in Fig. 5). Here, the slow flow rate (50 μl min^−1^) leads to a long residence time in the degassing unit, followed by a fast flow rate (600 μl min^−1^), which allows minimisation of the oxygen reabsorption in the tubing after the degassing unit. Similar slow-to-fast flow rate sequences can be applied when extremely low oxygen concentrations are required in small volumes and can be measured at stopped flow, for example, in determining the PL quantum efficiencies of oxygen-sensitive emitter molecules in solution.

### 3.6 Oxygen permeation in large-volume reactors

The permeation of gases through fluoropolymer tubes has been discussed, but stainless steel as tubing material could also be relevant for compatible chemical reactions. The primary disadvantage of stainless steel tubes compared to fluoropolymer tubes is that they are not acid-resistant. In addition, stainless steel tubes are very sturdy and thus lack the flexibility of polymer tubes.^43^ An advantage of the stainless steel tube is the lower oxygen permeability, which in turn should lead to lower oxygen reabsorption.^44^ This should especially be considered in ethereal solvents due to the risk of producing peroxides within the tubes in the presence of molecular oxygen.^45^

**Fig. 6:**
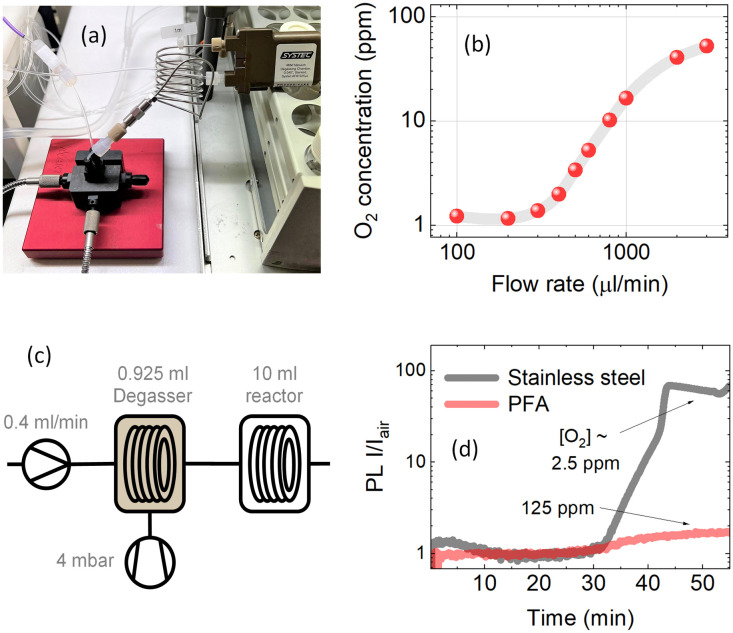
(a) Physical setup of the stainless 1 m steel tubing between the degassing chamber and the measurement point. (b) Oxygen concentration in PtOEP toluene solution at different flow rates. The oxygen concentration of the solution was measured after passing through a 1 m long 1 mm I.D. stainless steel tubing. The pressure of the degassing chamber was set to 4 mbar. (c) Updated schematic of the liquid degassing in continuous flow to include the 10 ml reactor. (d) The PL intensity ratio between the degassed solution and solution under ambient conditions over the time of the experiment. The results shown are for measurements after 10 ml of stainless steel tubing and 10 ml of PFA tubing, which take roughly 35 minutes at a flow rate of 400 μl min^−1^.

To test oxygen reabsorption in stainless steel tubing, the degassing unit was connected to the measurement cell with a 1 m long 1 mm I.D. stainless steel tube as shown in Fig. 6a. Oxygen concentration measurements as a function of solution flow rate showed almost no increase in oxygen content at slow flow rates <300 μl min^−1^ (see Fig. 6b). This indicates an almost negligible gas reabsorption after passing 1 m of steel tubing, allowing reaching full degassing capacity even in large-volume stainless steel reactors.

Here we demonstrate that in stainless steel reactors commonly used for high-temperature reactions in flow chemistry, a barrier for gas exchange with the environment is created. Fig. 6c shows a scheme, where a 5 μM PtOEP toluene solution was pumped at a constant 400 μl min^−1^ flow rate through a 925 μl degassing unit kept at 4 mbar vacuum pressure to reach approximately 1 ppm molar oxygen fraction before the solution was passed through a 10 ml reactor. Fig. 6d shows the comparison between the 10 ml PFA and stainless steel reactors tested under the same conditions. The almost 100-fold increase in PL intensity of the solution after passing through the stainless steel reactor indicated that the oxygen concentration in the solution was retained at the 2.5 ppm level even after more than 30 minutes of residence time in the reactor. In comparison, after passing through the 10 ml PFA reactor, the oxygen concentration in solution closely reached the ambient oxygen level due to the strong permeation of gases through the polymer tubing.

## 4 Conclusion

In this work, we present a method for efficient solvent and solution degassing and oxygen content monitoring in a continuous flow system based on a commercial degassing unit combined with a photoluminescence flow cell. We demonstrate that remote optical detection of photoluminescence quenching using micro-molar concentrations of oxygen-sensitive probes diluted in solvent is a highly sensitive technique to monitor below ppm-levels of oxygen in flowing liquids. Despite being rated at higher vacuum pressures, the commercial degassing unit showed robust and repeatable performance at 1–10 mbar vacuum pressures that allowed achieving oxygen concentrations 2–3 orders of magnitude lower than ambient oxygen concentrations in solvents under optimal conditions. Furthermore, the degassing system can be operated at hundreds of microliters per minute, which is relevant for performing oxygen-sensitive reactions in flow chemistry. Similarly, the flow degassing systems allow for automated measurements of oxygen-sensitive species without the burden of glovebox operation or inertisation procedures. A simple analytical model was developed and fitted to the experimental data. Using the model, it was possible to predict optimal flow rates to obtain the lowest possible oxygen concentrations at given lengths of tubing.

## Conflicts of interest

There are no conflicts to declare.

## Supplementary Material

RE-008-D3RE00109A-s001
