# Supplementary material for: Efficient degassing and ppm-level oxygen monitoring flow chemistry system
Source: React Chem Eng. 2023 Apr 28;8(8):2052–9. doi: 10.1039/d3re00109a (PMC10366651; doi:10.1039/d3re00109a)
Supplement: RE-008-D3RE00109A-s001 [file RE-008-D3RE00109A-s001.pdf]

Electronic Supplementary Material (ESI) for Reaction Chemistry & Engineering.
This journal is © The Royal Society of Chemistry 2023

Electronic Supplementary Information

Efficient degassing and ppm oxygen monitoring flow chemistry system

Paulius Baronas,[1] Jacob L. Elholm[1] and Kasper Moth-Poulsen[1, 2, 3, 4*]

[1] The Institute of Materials Science of Barcelona, ICMAB-CSIC, Bellaterra, 08193 Barcelona, Spain

[2] Catalan Institution for Research \& Advanced Studies, ICREA, Pg. Llu´ıs Companys 23, 08010 Barcelona, Spain

[3] Chalmers University of Technology, Department of Chemistry and Chemical Engineering. SE-412 96 Gothenburg, Sweden

[4] Department of Chemical Engineering, Universitat Politècnica de Catalunya, EEBE, Eduard Maristany 10–14, 08019 Barcelona, Spain

*corresponding author email: kasper.moth-poulsen@chalmers.se

Table of Contents

1. Evaluation of Stern-Volmer parameters 2
2. Effects of solvent back-pressure on degassing 3
3. Tube-in-tube nitrogen gas purging 4
4. Fits of the theoretical degassing model 5
5. Determination of experimental error 5

1. Evaluation of Stern-Volmer parameters

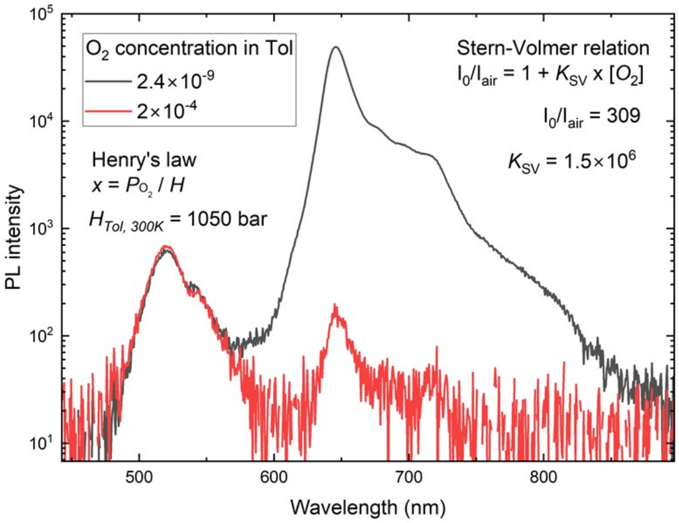

Figure S1. Photoluminescence intensity of 5 µM PtOEP in toluene (Tol) solution recorded at ambient O_2 concentration (20.9 %) (red line) and after 24 h degassing in glovebox with measured 2.4 ppm O_2 concentration (black line). Molar oxygen concentrations in solution were recalculated according to Henry's law and room temperature Henry's constant of 1050 bar. Photoluminescence intensity ratio (I_0/I_{air}) in degassed solution and air saturated solution and estimated Stern-Volmer constant (K_{SV}) are indicated.

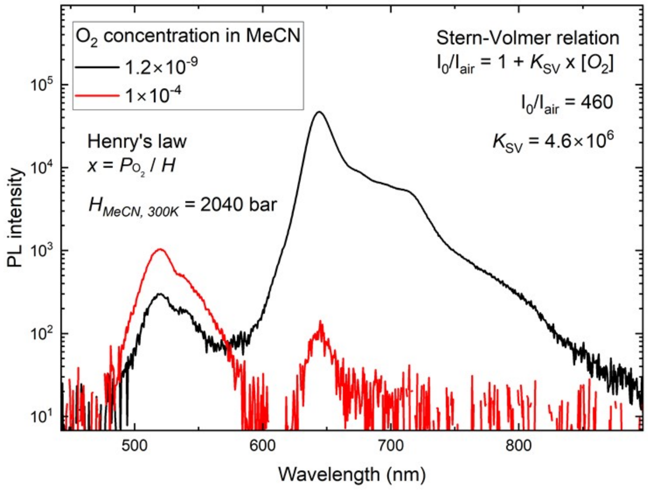

Figure S2. Photoluminescence intensity of 5 µM PtOEP in acetonitrile (MeCN) solution recorded at ambient O_2 concentration (20.9 %) (red line) and after 24 h degassing in glovebox with measured 2.4 ppm O_2 concentration (black line). Molar oxygen concentrations in solution were recalculated according to Henry's law and room temperature Henry's constant of 2040 bar. Photoluminescence intensity ratio (I_0/I_{air}) in degassed solution and air saturated solution and estimated Stern-Volmer constant (K_{SV}) are indicated. The variations of LED intensity at 515 nm were caused by scattering and not by different excitation intensity and thus can be neglected.

2. Effects of solvent back-pressure on degassing

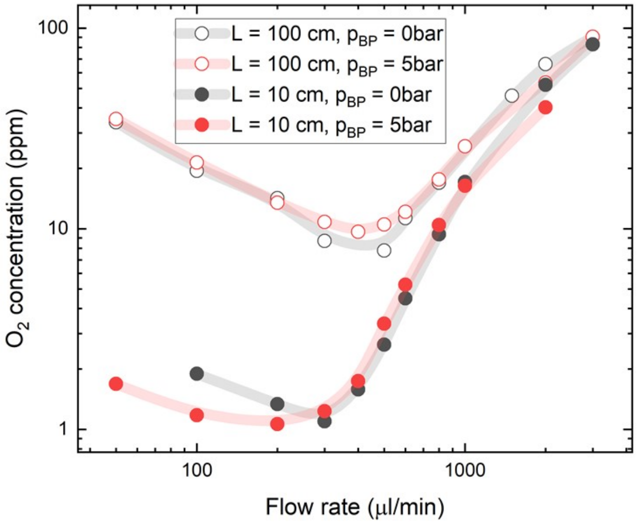

Figure S3. Oxygen concentration in PtOEP toluene solution as a function of solvent back-pressure (p_{BP}) and tubing length (L). Oxygen concentration was measured after single pass of ETFE tubing of 10 and 100 cm length at different solution flow rates. Vacuum pressure inside degassing chamber was kept at constant 4 mbar.

3. Tube-in-tube nitrogen gas purging

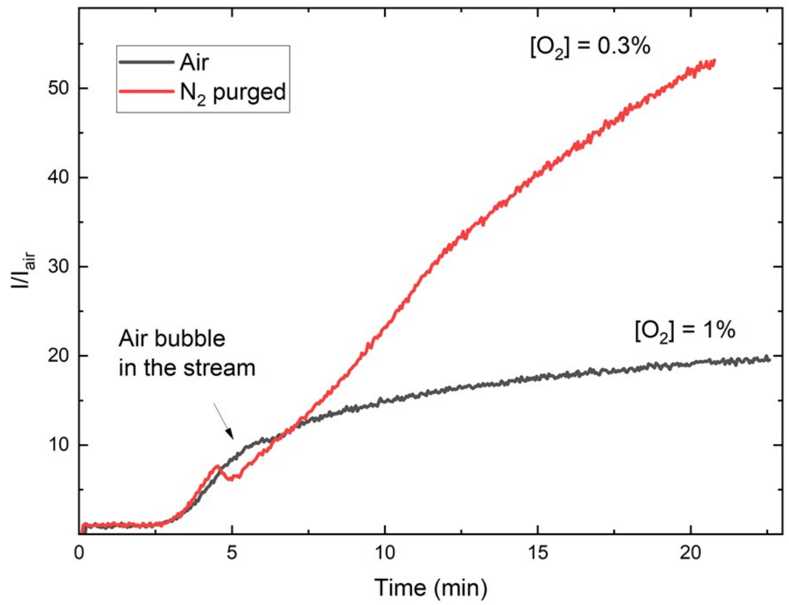

Figure S4. Photoluminescence intensity ratio improvement in N_2 purged compared to ambient air purged tube-in-tube reactor. PFA tubing (1 m length, 0.04” I.D., 1/16” O.D.) exiting degassing chamber was encased in PVC tubing (1 m length, 3 mm I.D.) with inlet and outlet for N_2 gas. Nitrogen gas pressure was kept at 40 mbar.

4. Fits of the theoretical degassing model

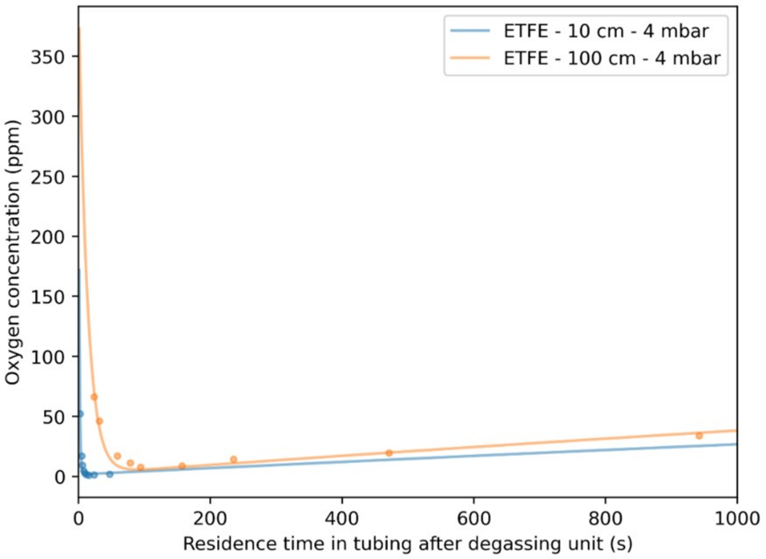

Figure S5. The fits of analytical solution to experimental data of toluene degassing at 4 mbar vacuum pressure. Residence time was calculated based on flow rate and tube volume after degassing unit with ETFE tubing (0.04” internal and 1/16” external diameter).

5. Determination of experimental error

Instrumental errors of optical oxygen detection technique include the accuracy of oxygen sensor inside glovebox, which was used for calibration of zero oxygen photoluminescence intensity (I_0), and the accuracy of spectrometer. The former error for MB-OX-SE1 oxygen sensor is set by oxygen concentration measurement accuracy of ±1 ppm in gaseous environment. This translates to error of determining zero oxygen photoluminescence intensity I_0/I_{air} = 309 ± 0.5 of PtOEP toluene solution. Similar error can be estimated for PtOEP in MeCN solution. The latter error is produced by noise of the spectrometer when measuring photoluminescence spectra. The standard deviation of the I/I_{air} signal was measured to be 0.2, which shows that at high I/I_{air} > 100 (low oxygen concentration) spectrometer error becomes negligible (< 0.2 %). Total instrumental error calculated for oxygen concentration is presented in the Figure S6. Low margin of error for determining oxygen concentration in solution can be explained by large dynamic range of PtOEP oxygen probe (I_0/I_{air} = 309) and high accuracy of MB-OX-SE1 oxygen sensor used for calibration.

There is also an error for setting flow rates determined by precision of peristaltic pumps (Vaportec V-3). Previously, pump calibration at low (0.25 ml/min) and high (2.5ml/min) flow rates revealed that set flow rates may vary up to ±10 % from the real flow rates. Therefore, error bars for the flow rate axis were also included, which shows significantly higher margin of error compared to measurement of oxygen concentration.

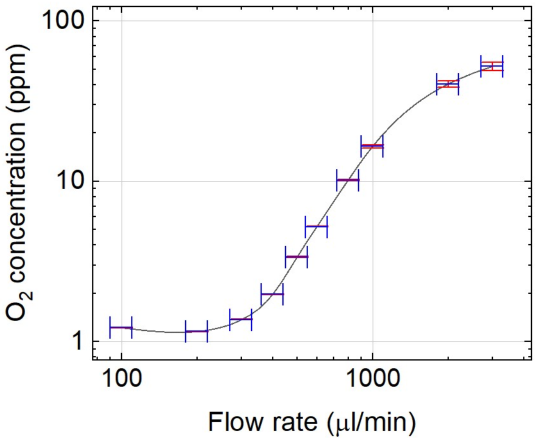

Figure S6. Error bars displayed for data of measured oxygen concentration in solution versus flow rate.
